# Household Hints and Recipes

**Published:** 1873-01

**Authors:** 


### Household Hints & Recipes.

WASHING HANDS WITH TURPENTINE.—Painters should seldom wash their hands in turpentine, as the practice, if persisted in, will lead to the most serious results, even to the loss of power in the wrist joints.

---

GOOD CEMENT.—A cement for fastening emery to wood may be prepared as follows: Melt together equal parts of shellac, white resin, and carbolic acid in crystals; add the last after the others are melted. The effect of the carbolic acid is surprising.

---

COLORED CHALK FOR TAILORS' USE.—Knead together ordinary pipe-clay, moistened, and ultramarine for blue, finely ground ochre for yellow, burnt ochre for red, etc., until they are uniformly mixed; roll out into thin sheets, cut, and press into wooden or metallic moulds well oiled to prevent sticking, and allow to dry slowly at ordinary temperature, or at a very gentle heat.

---

CHRONIC DIARRHŒA.—The following will be found to be an excellent remedy for chronic diarrhœa, where the usual remedies have failed. We have recommended it in numerous instances, and *always* with good results: One tablespoonful of arrowroot made into a thin gruel, with milk, and a tablespoonful of brandy added, taken morning and evening, before meals. Its effects are surprising, and the dose easy to take.

---

HOW TO SAVE COAL IN OPEN GRATES.—The *Spectator* remarks that "The most practical suggestion yet made towards economy of coals seems to be the use of solid bottoms in ordinary fire-grates. It is asserted, and indeed proved, that in any fire-place not excessively small, a plate of iron placed upon the grate will halve the consumption of coal, reduce the smoke, and leave a cheerful, free-burning fire. Quite sufficient air enters through the bars, no poking is necessary, and the fire never goes out till the coal is consumed. There is no ash and no dust, every particle of fuel being consumed. Any household can try this experiment, and reduce his coal bill, say, 20 per cent., at the cost of a shilling." This is not a new idea, but we believe it to be a good one.

METALLIC STAIN FOR WOOD.—Soaking the wood in a weak solution of nitrate of silver, and then exposing it to the light, will produce an intense black color. Another way is to boil some chips of logwood in water for about a quarter of an hour. Then wash the piece of wood with it three or four times, allowing it to dry after each washing. Lastly, wash the wood, by means of a common painting brush, with a mixture prepared as follows: Put one ounce of steel or iron filings into two ounces of vinegar, keep the phial near the fire so as to be gently heated for about two hours, then decant the vinegar, and keep it for use.—*Boston Journal.*

---

VARNISH TO IMITATE GROUND GLASS.—The *British Journal of Photography* gives the following recipe for this purpose: Dissolve ninety grains of sandarac and twenty grains of mastic in two ounces of washed methylated ether, and add, in small quantities, a sufficiency of benzine to make it dry with a suitable grain—too little making the varnish too transparent, and too much making it crapy. The quantity of benzine required depends upon its quality—from half an ounce to an ounce and a half, or even more; but the best results are got with a medium quantity. It is important to use washed ether free from spirit.

---

REPAIRING LEAKY ROOFS.—Melt together in an iron pot, two parts by weight of common pitch and one part of gutta-percha. This forms a homogenous fluid much more manageable than gutta-percha alone. To repair gutters, roofs, or other surfaces, carefully clean out of the cracks all earthly matters, slightly warm the edges with a plumber's soldering iron, then pour the cement, in a fluid state, upon the cracks while hot, finishing up by going over the cement with a moderately hot iron, so as to make a good connection and a smooth joint. The above will repair zinc, lead or iron, and is a good cement for aquariums.

---

ELASTIC VARNISH FOR LADIES' SHOES.—The *Manufacturer and Builder* gives this recipe:—

Three pounds of rain-water are placed in a pot over the fire, and when well boiling, there are added 4 ounces white pulverized wax, 1 ounce clear, transparent glue in small pieces, 2 ounces pulverized gum Senegal, 2 ounce white soap scraped fine, 2 ounces brown pulverized sugar; the ingredients are placed in one by one, and every time stirred up; it is well to take the pot from the fire every time a substance is added, to prevent boiling over; when all is added, the pot is removed from the fire; when sufficiently cooled, 3 ounces alcohol are added, and finally 8 ounces fine Frankport black, well incorporated by continued stirring. This varnish is put on the leather with a brush, and is very valuable for boots and shoes, as it can be afterwards polished with a large brush like ordinary shoeblacking, shows a high polish, and does not soil the clothing.

VERBENA WATER.—A good article may be prepared thus:—

| | |
|---|---|
| Rectified spirits | 1 pint. |
| Grass oil (verbena-oil) | 3 drachms. |
| Oil of lemon-peel | 2 ounces. |
| Oil of orange-peel | ½ ounce. |

Mix; let it stand for a few hours, filter if necessary, and fill in bottles. A very much superior article, also sold in commerce under the name of *Extrait de Verbene*, is made according to the following recipe:

| | |
|---|---|
| Rectified spirits | 1 pint. |
| Oil of orange-peel | 1 ounce. |
| Oil of lemon-peel | 2 ounces. |
| Oil of lemon | 1 drachm. |
| Grass-oil (verbena-oil) | 2½ drachms. |
| Ess. of orange-flowers | 7 ounces. |
| Essence of tuberose | 7 " |
| Essence of rose | ½ pint. |

—*Boston Jour. Chem.*

PULVERIZED COAL FOR UNHEALTHY PLANTS.—A correspondent of the *Revue Horticole* states that he brought a very fine rosebush, full of buds, and, after anxiously awaiting their maturing, was greatly disappointed to find the flowers small and of a dull, faded color. At the suggestion of a friend, he tried the experiment of filling in the top of the pot, around the bush, to the depth of half an inch, with finely pulverized hard coal. In a few days he was astonished at seeing the roses assume a hue as brilliant and lively as he could desire. He tried the same experiment upon a pot of petunias, and soon all the pale-colored ones became of a bright red or lilac, and the white ones were varigated with beautiful red stripes. Some of the lilac petunias became a fine dark blue. Other flowers experienced similar alterations; those of a yellow color alone remained insensible to the influence of the coal.

CURING HAMS.—The following recipes are those according to which the hams that took the first and second premiums at a Maryland State Fair were cured:—

1. Mix two and a half pounds of saltpetre finely powdered, one-half bushel fine salt, three pounds brown sugar, one-half gallon molasses. Rub the meat with the mixture; pack skin down. Turn over once a week, and add a little salt. After being down three or four weeks, take out, wash, and hang up two or three weeks until it is dry. Then smoke with hickory wood three or four weeks, then bag or pack away in a cool place—not a cellar—in chaff or hay.

2. The meat, after being cut out, must be rubbed, piece by piece, with very finely powdered saltpetre, on the flesh side, and when the leg is cut off, a table-spoonful (not heaped) to each ham, a dessert-spoonful to each shoulder, and about half that quantity to each middling and jowl; this must be rubbed in. Then salt it by packing a thin coating of salt on the flesh side of each piece, say one-half an inch thick; pack the pieces on a scaffolding, or on a floor with strips of plank laid a few inches apart all over it (that is, under the meat); the pieces must be placed skin side down, in the following order: First layer, hams; second, shoulders; third, jowls; fourth middlings—take the spare ribs out of the middling. The meat must lie in this wise six weeks if the weather is mild, eight if cold—the brine being allowed to run freely.—*Boston Jour. Chem.*

POISONOUS CONFECTIONERY.—If a report just presented to the Newcastle (England) town council had been available at the time Christiana Edmunds was on her trial, it might have been found useful in support of the theory of her lunacy. No one but a lunatic, it might have been urged, would take the trouble to poison confectionery and thereby incur suspicion, when there are, ready made to hand and openly sold in shops, sweetmeats artistically coated with deadly poison. That such is the case in Newcastle-upon-Tyne, is shown by a report of Mr. Pattison, analytical chemist, upon which the local corporation have decided to take immediate action. Mr. Pattison says he has examined various samples of sugar confectionary sold in Newcastle, and finds that nearly the whole of the articles colored yellow and orange are so colored by chromate of lead. Out of thirty-five different kinds of sweetmeats examined, obtained from twenty different dealers, twenty-eight were colored by this poison. Some of the articles contained upwards of a tenth of a grain of metallic lead, the engaging substance being supplied to manufacturers under the names of "lemon chrome" and "orange chrome." Mr. Pattison adds that "Some of the confectionery contained plaster of Paris to the extent of 1¾ per cent., besides a good deal of wheaten flour." If parents were allowed their choice, they would doubtless prefer the last named adulteration to the lead salt mentioned above.

---

HOUSE PLANTS IN WINTER.—Mr. James Vick, whose large experience as a florist makes him an unexceptionable authority on the subject, gives the following suggestions with regard to the management of house plants in winter :—

"Few plants can endure the high temperature and dry atmosphere of most of our living-rooms. The temperature should not be allowed to go above sixty-five in the day time, and not above forty in the night. As much air and light as possible should be given, while the leaves should be sprinkled every morning. A spare room, or parlor, or extra bedroom, is better for plants than a living-room. A bay window, connected with a warm room, especially if facing the south or east, makes an excellent place for keeping plants in winter. It should have glass doors on the inside, which can be closed a part of the time, especially when sweeping and dusting. The main thing in keeping house plants in health is to secure an even temperature, a moist atmosphere, and freedom from dust.—Sprinkle the leaves occasionally, and when they need water, use it freely. If the green fly, or aphis, appears, wash with soapsuds frequently, and occasionally with a little tobacco water, or a decoction of quassia chips. If the red spider comes, it shows the plants are in too dry an atmosphere. Burn a little sulphur under the plants, the fumes of which will kill the spider, and afterward keep the stems and leaves well moistened. Occasionally, but not often, worms appear in the pots. This can be avoided in a great measure by careful potting. A little weak lime water is sometimes of benefit in such cases, also five drops of liquid ammonia to a gallon of water, though, perhaps, the better way is to re-pot, removing the earth carefully, so as not to injure the growth of the plant."

